# Modeling the Formation of Transverse Weld during Billet-on-Billet Extrusion

**DOI:** 10.3390/ma7053470

**Published:** 2014-04-30

**Authors:** Yahya Mahmoodkhani, Mary Wells, Nick Parson, Chris Jowett, Warren Poole

**Affiliations:** 1 Mechanical and Mechatronics Engineering Department, University of Waterloo, 200 University Avenue West, Waterloo, ON N2L3G1, Canada; E-Mail: mawells@uwaterloo.ca; 2 Rio Tinto Alcan, Arvida Research and Development Centre, Jonquiere, QC G7S4L2, Canada; E-Mails: nick.parson@riotinto.com (N.P.); chris.jowett@sympatico.ca (C.J.); 3 Department of Materials Engineering, The University of British Columbia, 309-6350 Stores Rd, Vancouver, BC V6T1Z4, Canada; E-Mail: warren.poole@ubc.ca

**Keywords:** extrusion, transverse weld, finite element method, feeder, aluminum alloys

## Abstract

A comprehensive mathematical model of the hot extrusion process for aluminum alloys has been developed and validated. The plasticity module was developed using a commercial finite element package, DEFORM-2D, a transient Lagrangian model which couples the thermal and deformation phenomena. Validation of the model against industrial data indicated that it gave excellent predictions of the pressure during extrusion. The finite element predictions of the velocity fields were post-processed to calculate the thickness of the surface cladding as one billet is fed in after another through the die (*i.e.*, the transverse weld). The mathematical model was then used to assess the effect a change in feeder dimensions would have on the shape, thickness and extent of the transverse weld during extrusion. Experimental measurements for different combinations of billet materials show that the model is able to accurately predict the transverse weld shape as well as the clad surface layer to thicknesses of 50 μm. The transverse weld is significantly affected by the feeder geometry shape, but the effects of ram speed, billet material and temperature on the transverse weld dimensions are negligible.

## 1. Introduction

Billet-on-billet extrusion is utilised in the vast majority of commercial aluminum extrusion operations. This is done to allow use of sophisticated handling systems that maximize productivity of the extrusion process. On the other hand there are unwanted consequences of extruding in this manner including the development of a transverse weld which will develop between the individual billets as they are extruded into the final extrudate. This can result in a profile that does not meet the mechanical property specification. In addition, the surface of the second billet is clad with the first billet with remnants of the oxide films from the billet end and feeder face on the interface. This effect can extend for many metres into the second billet and the main objectives in continuous billet-on-billet extrusion is to minimize the transverse weld interface length and the associated scrap allowance, while at the same time providing a weld strong enough to withstand the stretching process after extrusion. It has been shown that the transverse welding length depends on how fast the material from the old billet flows out at the die corners [1]. Consequently, the geometry of the feeder and die can significantly affect the length and thickness of the transverse weld in the extrudate [2,3]. It is now recognised [3] that in addition to the physical effects of the transverse weld interface on product integrity, material from successive billets can accumulate inside in the feeder pocket and gradually enter the product surface generating mill finish or post-anodised streaks. Therefore feeder pocket geometry plays a key role on many aspects of extruded product quality and the ability to predict the effect of changes in feeder design is a critical step in the improvement of press recovery.

Using a validated mathematical model of the extrusion process, this research shows the effects of parameters such as billet material, feeder geometry and extrusion ratio on the material flow and the extent of the transverse welds in the extrudate.

### Transverse Weld Formation

During the extrusion process, sticking friction usually occurs between the billet and container which results in inhomogeneous flow of material towards the die, such that the centre of the billet flows towards the die faster than the billet surface [4,5]. When extruding more than one billet, the extrusion is stopped before the back-end defect can occur, the remaining part of the billet is sheared off and a new billet is loaded into the container. The two billets then butt up against each other and they essentially become “welded” together as the extrusion continues and the billet surfaces are forced together. In this billet-on-billet extrusion process, which is often used in modern aluminum extrusion plants as a mean to maintain continuous production, transverse welding of the two billets can occur; the billet surfaces are forced together under high pressure as the ram pushes them towards the feeder and into the die. Due to the material flow pattern, as the billet enters the feeder and die, the interface (transverse weld) between the billets does not remain flat and can manifest itself on the extrudate as a clad surface layer [6]. Jowett *et al.* [3] found that the thickness of the transverse weld over the length of extrudate fits well with a power law function.

**Figure 1.**
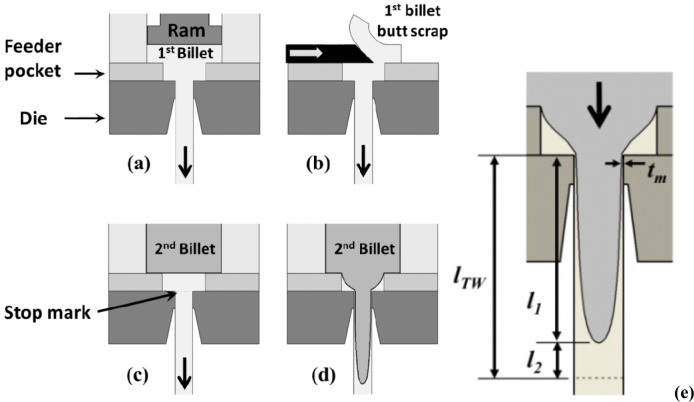
Schematic of transverse weld formation showing: (**a**) end of the extrusion of the first billet; (**b**) shearing the 10% billet butt off the first billet; (**c**) loading the second billet into the container; (**d**) beginning of extrusion of the second billet, showing the development of the transverse weld; (**e**) the length of the transverse weld (*l_TW_*) with minimum clad thickness of *t_m_* divided into *l*_1_ and *l*_2_.

Figure 1 shows an illustration of the formation of a transverse weld or clad surface layer between two consecutive billets. During the press stop when the butt is sheared and the next billet loads, a visible mark forms where the die bearing contacts the profile. This mark, known as the “stop mark”, can be distinguished on the surface after extrusion.

**Figure 2.**
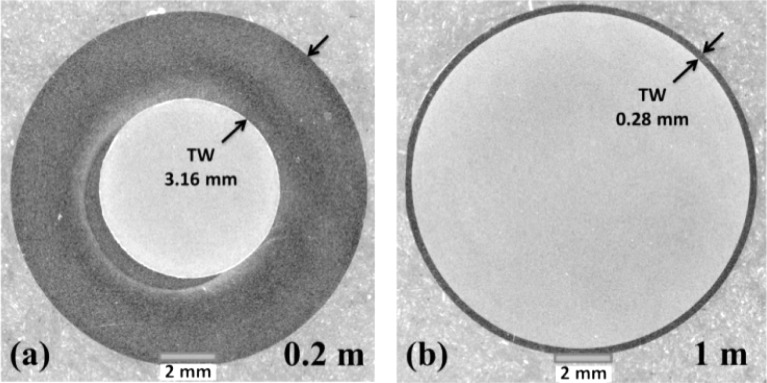
Optical micrograph showing the cross section of transverse weld at (**a**) 0.2 m and (**b**) 1 m from stop mark for an extrusion ratio of 70 (first billet AA6063 and second billet AA3003).

Figure 2 shows cross-section images of transverse weld (black ring) at the start (a) and the middle of transverse weld (b). As shown in Figure 2a, at the distance of 0.2 m from stop mark the thickness of the clad layer is 3.16 mm and at the distance of 1 m, Figure 2b, the transverse weld is 0.28 mm.

## 2. Results and Discussion

### 2.1. Dead Metal Zone and the Effect of Feeder Geometry

**Figure 3.**
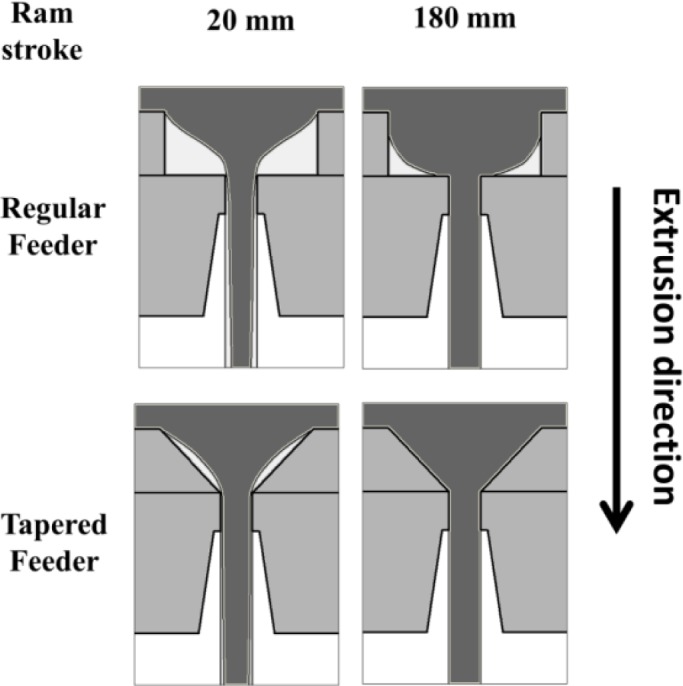
Effect of feeder geometry on shape of transverse weld at different ram strokes, dotted lines at the first snapshot indicate the position of the stop mark. (Model predictions for extrusion ratio of 70; billet temperature = 500 °C; ram speed = 2 mm/s).

Figure 3 shows the model-predicted effect of feeder geometry on the material flow and dead metal zone during a round bar extrusion with extrusion ratio of 70. The first snapshot on the left is for a ram stroke of 20 mm which is 4 mm after the break through. During the entire extrusion, the previous billet material left inside the regular feeder geometry is much larger than that of the tapered feeder. This arises from both the lower initial volume of material remaining inside the tapered feeder from the first billet as well as the different metal flow pattern. At the end of the extrusion (ram stroke of 180 mm for this case), there is no prior billet material left in the tapered feeder but there is some prior billet material left inside the regular feeder.

### 2.2. Evaluation of Transverse Weld Thickness

**Figure 4.**
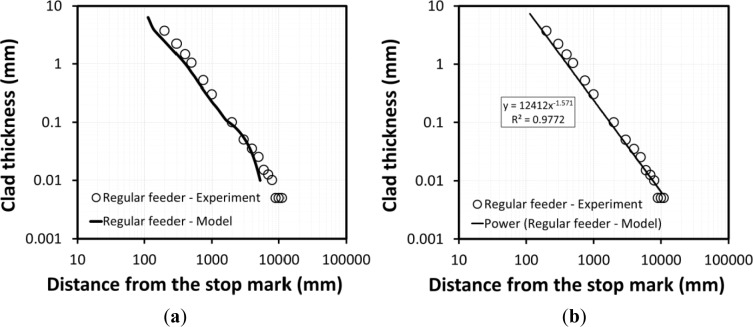
(**a**) Model predictions and experimental measurements for transverse weld thickness plotted on logarithmic scales; (**b**) power law trendline fitted on model predictions, (first and second billet AA3003 with homogenization of 24H600 and 8H550, respectively).

To validate the model predictions of the geometry of the transverse weld, the thickness of the surface clad layer from the previous billet was measured at given positions and compared to model predictions. Figure 4 shows a comparison of model predictions of the clad layer against experimental measurements for transverse weld thickness along the extrudate. For this feeder configuration and extrusion ratio, residual material from the previous billet was present along the entire length of the extruded profile. The model predictions of the clad layer thickness fit well with power law trend line with an exponent of about −1.5 which was earlier reported by Jowett *et al.* [3] for simple rectangular and shaped feeder plate geometries. As shown in Figure 4a, the model predictions are in good agreement with experimental measurements until the thickness of clad layer falls below ~30 μm where the model predictions deviate from the experimental measurements. This is probably due to the mesh size used near the surface (~100 μm); with smaller mesh size the model predictions would most likely be more accurate to lower clad thicknesses. Figure 4b shows that the trendline fit on the model predicted data agrees well with the experimental data throughout the extrudate, even at very low clad thickness where the model shows some deviation from the experimental measurements.

### 2.3. Effect of Billet Material

**Figure 5.**
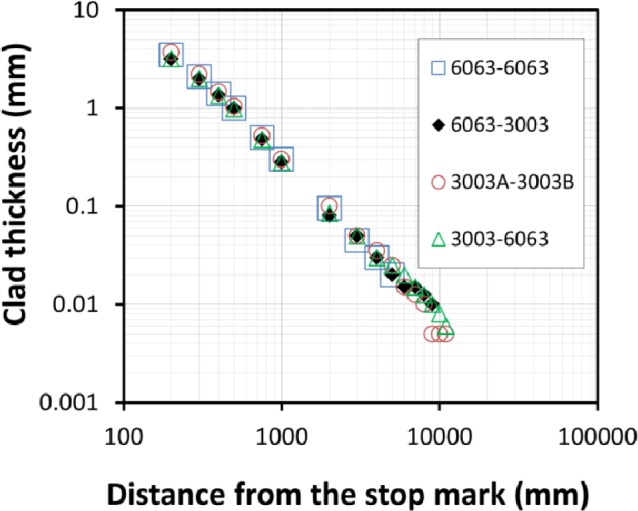
Effect of different billet material combination on the thickness of transverse weld along the extrudate (legends represent the codes for aluminum alloy used for first and second billets for each test, 3003A stand for 3003 aluminum alloy homogenized at 600 °C for 24 h and 3003B stands for the same alloy homogenized at 550 °C for 8 h).

**Figure 6.**
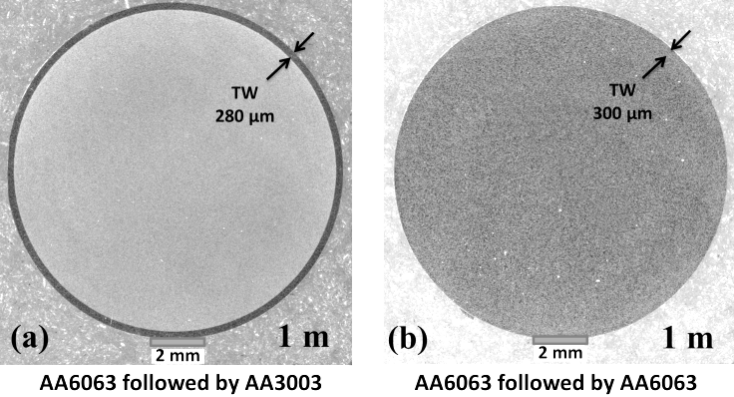
Comparison of transverse weld contrast (**a**) for marker materials and (**b**) regular AA6063 at 1000 mm position.

The effect of different combinations of alloy types and homogenisation practices on the transverse weld shape is shown in Figure 5. The effect of billet material on the shape of the transverse weld appears to be negligible at least for the AA3XXX and AA6XXX alloys studied. This indicates that the model predictions done on clad thickness should be applicable to a range of aluminum alloys. Normally transverse scrap allowances are confirmed by macroetching the profile cross-section and determining the position at which the defect is no longer visible by the naked eye or low power microscope. In these tests the thickness of the residual material from the previous billet was measured by optical metallography on polished sections. When marker materials were used, the contrast between alternate billets was clear as shown in Figure 6a which allowed very thin layers <20 μm in thickness to be identified along the entire profile length. In Figure 5 it is interesting to note that for AA6063 followed by AA6063 (large open squares) the metallographic examination only identified the transverse weld extending to 5 m, *i.e.*, half the extruded length. As shown in Figure 6b, even at the 1 m position for AA6063 the contrast associated with the transverse weld was not clear compared with the marker material situation. As indicated by the trends in Figure 5 there is no reason why the weld length for AA6063 should be any shorter. In fact the original metallographic work only identified transverse weld extending to 1,000 mm for AA6063 and based on the results for the marker materials the samples were re-examined and traces of transverse weld were found at the 5 m position. This result is not surprising as, for the same material, observation of the transverse weld interface relies on resolution of the oxide film from the billet end surface which is progressively broken up as the weld progresses. For structural integrity the avoidance of the oxide layer is the main issue and normal practices usually ensure this is not incorporated in saleable product. However, this result does indicate that standard practices for determining scrap allowances typically underestimate the presence of residual material in surface layers <100 μm in thickness. This suggests that most commercial products have a surface layer at least partially composed of the preceding billet which has implications for streak formation.

### 2.4. Effect of Feeder Geometry

**Figure 7.**
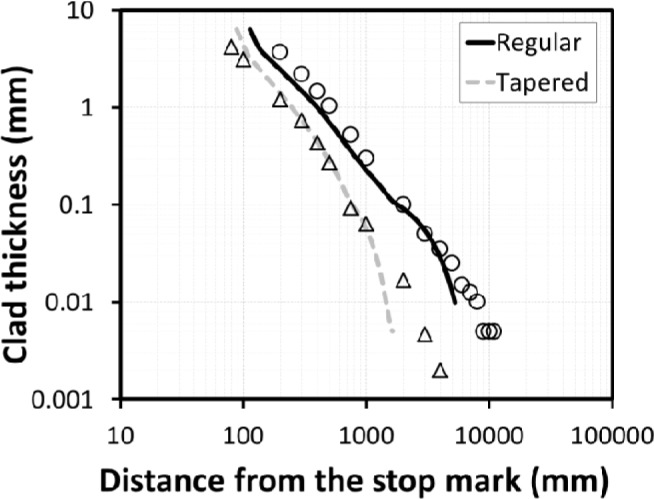
Comparison of model predictions (lines) to measurements (symbols) showing the effect of feeder shape on transverse weld geometry for extrusion ratio of 70 (circles and triangles represent measurements for regular and tapered feeder, respectively).

Figure 7 shows the model predictions and measurements for the effect of tapered feeder on the thickness and length of transverse weld. It is observed that using a tapered feeder significantly decreases the length of product contaminated by the transverse weld defect and the clad thickness.

## 3. Experimental Procedure

A series of extrusion trials were conducted using the fully instrumented pilot-scale extrusion press at the Rio Tinto Alcan Arvida Research and Development Centre located in Jonquiere, Quebec. This is a 780 t direct extrusion press with a 106 mm diameter container. The press is highly instrumented. The main ram position is measured using a Baluff digital position transducer and this information is used to calculate extrusion speed. Extrusion force is determined by the hydraulic pressure acting on the main and side cylinders. Billet temperature is accurately measured by three contact thermocouples located on the billet loading clamp. The container liner contains 12 thermocouples in holes drilled through the container liner such that they touch the liner surface.

AA3003 billets were DC-cast into 101.6 mm diameter billets with lengths of 200–400 mm and then homogenized at three different homogenization conditions. Table 1 shows the range of conditions tested during the extrusion trials. For all trials listed here a 60 mm diameter feeder plate was used.

To determine the effect of billet material on the transverse weld and also to see a good contrast between the clad layer and the extrudate, two billets with different material (AA6063) were extruded in between the other billets.

Table 2 shows the chemical composition of the AA3003 and AA6063 aluminum alloys used in this study.

Prior to extrusion, the billets were rapidly reheated in an induction furnace to the extrusion temperature, and extruded into round bars.

During the extrusion of each billet, the ram was stopped when 10% of the original billet length remained in the container. The ram and container were then moved back to make it possible to shear the butt scrap. Typically the butt or discard length is set as a percentage of the original billet length. In our case with a starting billet length of 200 mm, 20 mm of butt scrap was left. The extrusion process then continues once the container is repositioned and the next billet loaded.

To measure the thickness of the transverse weld, the extrudate was sectioned at different positions, polished and etched for 60 s with HF 0.5% solution and digital images were taken using the optical microscope with variable lighting filter to see the contrast between the clad layer and the extrudate.

**Figure 8.**
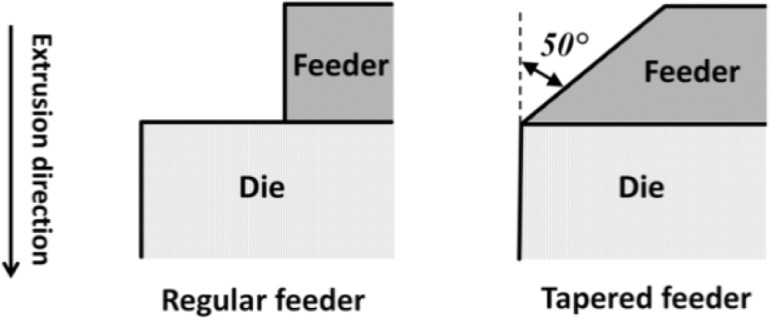
Schematic of two different feeder geometries.

One additional extrusion trial was performed using a tapered feeder to investigate the effect of feeder geometry on the length of the transverse weld. While this type of geometry is not commonly used for aluminum extrusion, it was selected to impose a major change in the material flow during extrusion. The maximum inner diameter of the tapered feeder was 60 mm (equal to the diameter of regular feeder) and its exit diameter was 12.7 mm (the same as the die diameter). In both cases, the feeder depth was 25 mm. Figure 8 provides a schematic of the two feeder geometries used.

## 4. Mathematical Model

### 4.1. Finite Element Model

The commercial finite element (FE) package; DEFORM-2D; was used to numerically model the extrusion process. The code is based on the flow formulation approach using an updated Lagrangian procedure. The 2D axisymmetric geometry was divided into a series of four-node; quadrilateral; linear and iso-parametric elements. These FE element types were the only ones available in the version of DEFORM-2D (Version 10) used for these simulations. The calculation time to simulate extrusion of the entire billet using a minimum element size of 0.25 mm was about 8 days; using a 3rd generation Intel i7 processor. More detailed information on the FE model is described in a previous paper [7].

Since there is large plastic deformation associated with the extrusion process, the billet material was assumed to behave as a rigid-viscoplastic material. Whereas the other objects were defined as being rigid during deformation; only the billet was involved in the deformation to which the flow formulation analysis applies. In this work the solute drag based constitutive model developed by Kocks and Chen [8], shown in Equation (1), was used to capture the temperature and strain rate dependence of the material flow stress.

$$\dot{\varepsilon }=A{\left(\frac{\sigma }{G}\right)}^{n}\frac{Gb^{3}}{kT}\text{exp}(-\frac{Q_{d}}{RT})$$ (1)

where, $\dot{\varepsilon }$ is the strain rate; is the stress; *G* is the temperature dependent shear modulus; *b* is the temperature dependent magnitude of the Burgers vector; k is Boltzmann’s constant; *T* is the deformation temperature; *Q*_d_ is the activation energy for diffusion for the rate controlling diffusing species (in this case Mn); R is the gas constant; and A and n are material constants that were determined from experimental studies and were found to depend on the starting chemistry and homogenization treatment [9].

**Figure 9.**
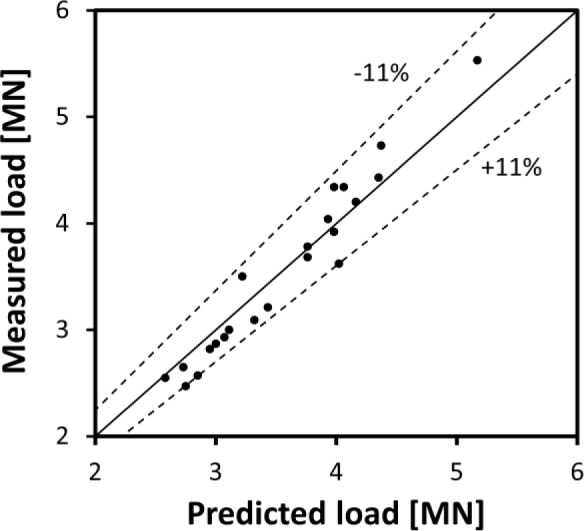
Comparison of predicted and measured extrusion load for different conditions listed in Table 1. Load measurements were taken at the ram stroke which coincided when 50% of the billet length was extruded.

Figure 9 illustrates the capability of the 2D FEM model to predict the extrusion load over the range of extrusion conditions outlined in Table 1, by comparing the predicted and measured loads at a ram position of 50% of the billet length. As shown in the figure, the model predicted load falls within ±11% of the measured load, with the average difference of −1.4% and average absolute difference of 5.3%.

### 4.2. Post Processing of FEM Results

**Figure 10.**
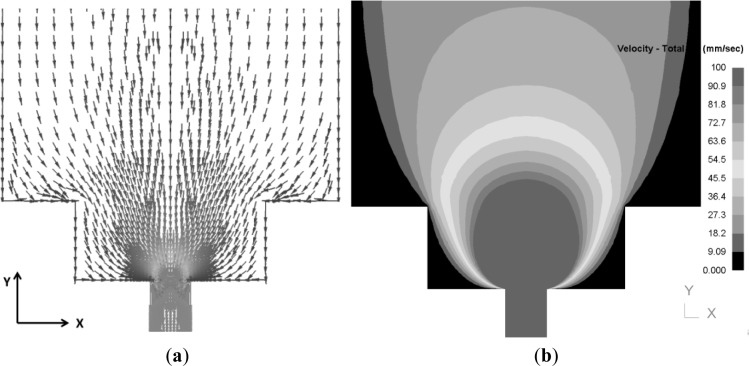
FE model-predicted velocity field showing (**a**) the direction and (**b**) the magnitude of velocity through the deforming material (extrusion ratio = 70, arrows show the direction of velocity for each node).

Using MATLAB software, results for velocity flow fields from the FEM model were processed so that details of the flow pattern and transverse weld thickness could be determined. The first step in the post processing of the FEM results was tracking different points through the velocity field from the billet during extrusion and into the final extrudate. Figure 10 shows the direction and magnitude of velocity for each nodal point in the billet for an extrusion ratio of 70.

Predictions of the transverse weld shape and thickness were made by tracking the points of the boundary surface between the two billets. Details of the mathematical procedure for point tracking and determination of transverse weld thickness are presented in a previous paper [7].

## 5. Conclusions

Using a simple but effective numerical technique in conjunction with FE model predictions, the thickness and length of the transverse weld was accurately calculated for radically different feeder geometries giving good agreement with physical measurements. Although the calculations were done predominantly on AA3XXX aluminum alloys, these predictions would be applicable to other aluminum alloy systems.

For round bar extrusion using a simple cylindrical feeder, the simulations indicated that material from the preceding billet is still present in the dead zones of the pocket such that the surface of the product can be contaminated over the entire length. In the case of the tapered pocket, material from the preceding billet clears from the pocket during the push, significantly decreasing the transverse weld length.

Experimental measurements and model predictions indicate that the thickness of the clad layer as a function of extrudate position fits well with a power law with an exponent of −1.6.

The transverse weld length did not vary with the changes in billet material made in the study. However, using dissimilar materials did allow the depth of the surface layer to be tracked to <100 μm, indicating that the normal methods industrially used to reveal the transverse weld disregard the presence of thin surface layers.

## Figures and Tables

**Table 1.** Range of extrusion conditions studied.

Extrusion parameter		Value	
Extrusion ratio	17	70	280
Temperature (°C)	400, 500	400, 450, 500	400, 500
Ram speed (mm/s)	8	2, 8, 20, 32	2, 8
Billet length (mm)	400	200	200
Homogenization *	8H550	8H500, 8H550, 24H600	8H550

* 8H500 stands for 8 h at 500 °C and so on.

**Table 2.** Weight percentage of major alloying elements in aluminum alloys used for the billet material.

Types	Weight percentage of major alloying elements (wt%)			
	Mn	Fe	Si	Mg
AA3003	1.27	0.54	0.10	–
AA6063	0.035	0.17	0.44	0.5

## References

[1] Li Q., Harris C., Jolly M.R. (2003). Finite element modelling simulation of transverse welding phenomenon in aluminium extrusion process. Mater. Des.

[2] Hatzenbichler T., Buchmayr B. (2010). Finite element method simulation of internal defects in billet-to-billet extrusion. J. Eng. Manuf.

[3] Jowett C., Parson N., Guay R., Fafard S., Maltais A. The Dynamics of the Dead Zones in Hot Extrusion.

[4] Donati L., Tomesani L., Schikorra M., Khalifa N.B., Takkaya A.E. (2010). Friction model selection in FEM simulations of aluminium extrusion. Int. J. Surf. Sci. Eng.

[5] Mahmoodkhani Y., Wells M.A., Parson N., Geng Y., Poole W.J. Mathematical Modelling of the Extrusion of AA3XXX Aluminum Alloys.

[6] Sheppard T. (1999). Extrusion of Aluminium Alloys.

[7] Mahmoodkhani Y., Wells M.A., Parson N., Poole W. (2014). Numerical modelling of the material flow during extrusion of aluminium alloys and transverse weld formation. J. Mater. Process. Technol.

[8] Kocks U.F., Chen S.R. Constitutive Laws for Deformation and Dynamic Recrystallization in Cubic Metals.

[9] Kubiak A. (2009). Effect of Homogenization on High Temperature Deformation behaviour of AA3XXX Aluminum Alloys. Master’s Thesis.

